# *catena*-Poly[[[diaqua­iron(II)]-μ-pyridine-2,5-dicarboxyl­ato-[tetra­aqua­iron(II)]-μ-pyridine-2,5-dicarboxyl­ato] tetra­hydrate]

**DOI:** 10.1107/S1600536808002043

**Published:** 2008-01-25

**Authors:** Hai-Yun Xu, Huai-Ling Ma, Mao-Tian Xu, Wen-Xian Zhao, Bao-Guo Guo

**Affiliations:** a Department of Chemistry, Shangqiu Normal College, 476000 Shangqiu, Henan, People’s Republic of China

## Abstract

In the crystal structure of the title compound, {[Fe_2_(C_7_H_3_NO_4_)_2_(H_2_O)_6_]·4H_2_O}_*n*_, there are two types of coordination for the Fe^II^ atoms. One Fe^II^ atom is in a distorted octa­hedral N_2_O_4_ environment, with two chelating rings from the pyridine­dicarboxyl­ate ligands and two O atoms from the water mol­ecules, while the other is in a distorted octa­hedral O_6_ environment with two O atoms from the pyridine­dicarboxyl­ate ligands and four O atoms from the water mol­ecules. Both Fe^II^ atoms lie on crystallographic centers of symmetry. The complex possesses an infinite chain structure running along the [101] direction. These chains are inter­connected by the uncoordinated water mol­ecules through O—H⋯O hydrogen bonds.

## Related literature

For related literature, see: Hill (1998 ▶); Liang *et al.* (2001 ▶); Mitzi *et al.* (1995 ▶); Moler *et al.* (2001 ▶); Zeng *et al.* (2003 ▶); Xu *et al.* (2004 ▶).
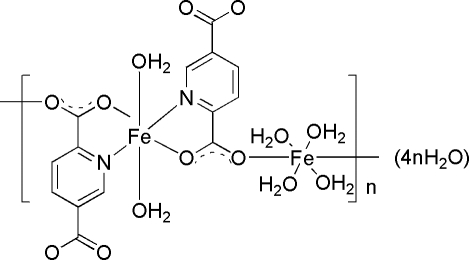


## Experimental

### 

#### Crystal data

[Fe_2_(C_7_H_3_NO_4_)_2_(H_2_O)_6_]·4H_2_O
*M* *_r_* = 622.06
Triclinic, 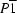

*a* = 7.098 (3) Å
*b* = 8.922 (3) Å
*c* = 9.720 (2) Å
α = 90.942 (6)°
β = 101.375 (6)°
γ = 108.112 (5)°
*V* = 571.6 (3) Å^3^
*Z* = 1
Mo *K*α radiation
μ = 1.36 mm^−1^
*T* = 298 (2) K
0.21 × 0.20 × 0.18 mm

#### Data collection

Bruker SMART APEX CCD diffractometer
Absorption correction: multi-scan (**SADABS**; Sheldrick, 1996 ▶) *T*_min_ = 0.763, *T*_max_ = 0.792
2866 measured reflections
1989 independent reflections
1757 reflections with *I* > 2σ(*I*)
*R*_int_ = 0.062

#### Refinement

*R*[*F* ^2^ > 2σ(*F* ^2^)] = 0.043
*wR*(*F* ^2^) = 0.124
*S* = 1.06
1989 reflections
166 parameters
H-atom parameters constrained
Δρ_max_ = 0.65 e Å^−3^
Δρ_min_ = −0.60 e Å^−3^

### 

Data collection: *SMART* (Siemens, 1996 ▶); cell refinement: *SAINT* (Siemens, 1996 ▶); data reduction: *SAINT*; program(s) used to solve structure: *SHELXS97* (Sheldrick, 2008 ▶); program(s) used to refine structure: *SHELXL97* (Sheldrick, 2008 ▶); molecular graphics: *SHELXTL* (Sheldrick, 2008 ▶); software used to prepare material for publication: *SHELXTL*.

## Supplementary Material

Crystal structure: contains datablocks global, I. DOI: 10.1107/S1600536808002043/is2270sup1.cif

Structure factors: contains datablocks I. DOI: 10.1107/S1600536808002043/is2270Isup2.hkl

Additional supplementary materials:  crystallographic information; 3D view; checkCIF report

## Figures and Tables

**Table 1:** Hydrogen-bond geometry (Å, °)

*D*—H⋯*A*	*D*—H	H⋯*A*	*D*⋯*A*	*D*—H⋯*A*
O9—H9*B*⋯O8	0.85	2.32	3.159 (6)	171
O9—H9*A*⋯O6^i^	0.85	2.06	2.849 (5)	154
O8—H8*B*⋯O5^ii^	0.85	2.55	3.204 (4)	134
O8—H8*B*⋯O4^ii^	0.85	2.51	3.171 (4)	136
O8—H8*A*⋯O3	0.85	2.55	3.177 (4)	132
O8—H8*A*⋯O2	0.85	2.44	3.201 (4)	149
O5—H5*B*⋯O7^i^	0.85	2.22	2.706 (3)	116
O2—H2*B*⋯O9^iii^	0.85	1.94	2.657 (5)	141
O2—H2*A*⋯O4^iv^	0.85	1.99	2.758 (3)	150
O1—H1*B*⋯O6^v^	0.85	1.92	2.715 (3)	156
O1—H1*A*⋯O8^ii^	0.85	2.06	2.822 (4)	148

Symmetry codes: (i) 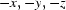; (ii) 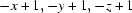; (iii) 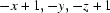; (iv) 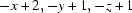; (v) 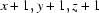.

**Fig. 1.**
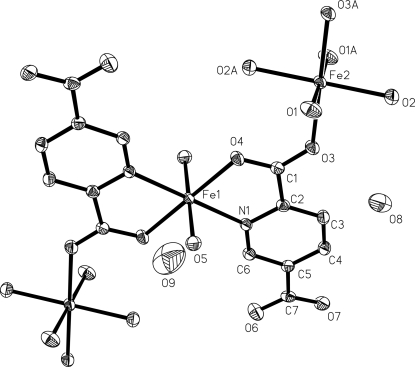
Part of the polymeric structure of the title compound, showing 30% probability displacement ellipsoids and the atom-numbering scheme. The H atoms are omitted for clarity. The suffix A corresponds to symmetry code (2 - x, 1 - y, 1 - z).

**Fig. 2.**
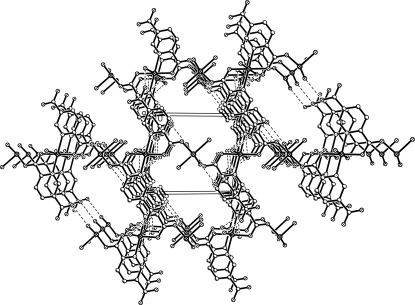
The three-dimensional supramolecular structure of the title compound. Hydrogen bonds are shown by dashed lines.

## supplementary crystallographic information

### Comment

Extended frameworks of coordination polymers based on transition metal ions and multifunctional bridging ligands are currently of great interest because of their intriguing topologies and their potential applications (Hill, 1998; Moler *et al.*, 2001; Mitzi *et al.*, 1995). Multi-carboxylate ligands may exhibit various coordination modes to furnish various structures. Recently, many transition metal-organic polymers constructed with multi-carboxylate ligands show varies of novel topology and potential applications in catalysis, materials chemistry and biochemistry (Zeng *et al.*, 2003; Xu *et al.*, 2004; Liang *et al.*, 2001). Pyridine-2,5-dicarboxylic acid (H_2_pydc) has unique features because of the presence of two carboxylate groups (O donor atoms) and the pyridine ring (N donor atom), which aids to increase the dimensionality of the assembled covalent network. Therefore, it is most likely that pydc will form low symmetric structures with metals. In this paper, we report the preparation and crystal structure of a new three-dimensional supramolecular complex [Fe_2_(pydc)_2_(H_2_O)_2_].4H_2_O, (I).

In the complex, (I), there exist two types of coordination geometries around the Fe(II) ions. The Fe1 ions are hexacoordinated in a N_2_O_4_ environment with two chelating rings from the pydc ligands and two oxygen atoms from the water molecules. The Fe2 ions are also hexacoordinated in an O_6_ environment with two oxygen atoms from the pydc ligands and four oxygen atoms from the water molecules. All Fe atoms lie on a crystallographic center of symmetry and the ligand lies on a crystallographic twofold axis. A perspective view of the local coordination environments around the Fe(II) atoms of (I) is shown in Fig. 1. For Fe1 and Fe2, the bond distances of Fe—O (water oxygen), 2.071–2.100 Å, are similar with those of Fe—O (carboxylate oxygen), 2.058–2.080 Å. As presented in Fig. 2, the two kinds of geometries around Fe(II) ions are arranged alternatively to give the one-dimensional polymeric chain. Interestingly, all Fe atoms of one polymeric chain are situated on one line and the neighboring Fe(II) atoms are *syn*-anti carboxylato bridged with the distance of 5.423 Å. These chains are interconnected by the uncoordinated water molecules through O—H···O hydrogen-bonding interactions and form a two-dimensional layer structure. A three-dimensional supramolecular network is obtained through O—H···O hydrogen-bonding interactions in the layers.

### Experimental

A mixture of H_2_pydc (0.34 g, 2 mmol), KOH (0.23 g, 4 mmol) and FeSO_4_.7H_2_O (0.55 g, 2 mmol) in 15 ml of MeOH/H_2_O (*v*/*v*, 1:1) was sealed in a 25-ml stainless-steel reactor with a teflon liner and was heated at 453 K for 72 h under autogenous pressure. Slow cooling to room temperature yielded 0.36 g (yield 40%) of block red crystals. Anal. Calc. for C_7_H_13_NO_9_Fe (%): C 27.03, H 4.21, N 4.50. Found (%): C 27.14, H 4.46, N 4.36.

### Refinement

The H atoms were included in the riding-model approximation with C—H = 0.93 Å and O—H = 0.85 Å, and with *U*_iso_(H) = 1.2*U*_eq_(C, O). Hydroxyl H atoms were allowed to rotate to best fit the experimental electron density.

### Crystal data

**Table:**

[Fe_2_(C_7_H_3_NO_4_)_2_(H_2_O)_6_]·4H_2_O	*Z* = 1
*M**_r_* = 622.06	*F*_000_ = 320
Triclinic, *P*1	*D*_x_ = 1.807 Mg m^−^^3^
Hall symbol: -P 1	Mo *K*α radiation λ = 0.71073 Å
*a* = 7.098 (3) Å	Cell parameters from 714 reflections
*b* = 8.922 (3) Å	θ = 2.4–28.2º
*c* = 9.720 (2) Å	µ = 1.36 mm^−^^1^
α = 90.942 (6)º	*T* = 298 (2) K
β = 101.375 (6)º	Block, red
γ = 108.112 (5)º	0.21 × 0.20 × 0.18 mm
*V* = 571.6 (3) Å^3^	

### Data collection

**Table:**

Bruker SMART APEX CCD diffractometer	1989 independent reflections
Radiation source: fine-focus sealed tube	1757 reflections with *I* > 2σ(*I*)
Monochromator: graphite	*R*_int_ = 0.062
*T* = 298(2) K	θ_max_ = 25.0º
φ and ω scans	θ_min_ = 2.1º
Absorption correction: multi-scan(*SADABS*; Sheldrick, 1996)	*h* = −8→6
*T*_min_ = 0.763, *T*_max_ = 0.792	*k* = −10→10
2866 measured reflections	*l* = −11→10

### Refinement

**Table:**

Refinement on *F*^2^	Secondary atom site location: difference Fourier map
Least-squares matrix: full	Hydrogen site location: inferred from neighbouring sites
*R*[*F*^2^ > 2σ(*F*^2^)] = 0.043	H-atom parameters constrained
*wR*(*F*^2^) = 0.124	*w* = 1/[σ^2^(*F*_o_^2^) + (0.081*P*)^2^ + 0.2244*P*] where *P* = (*F*_o_^2^ + 2*F*_c_^2^)/3
*S* = 1.06	(Δ/σ)_max_ < 0.001
1989 reflections	Δρ_max_ = 0.65 e Å^−^^3^
166 parameters	Δρ_min_ = −0.60 e Å^−^^3^
Primary atom site location: structure-invariant direct methods	Extinction correction: none

### Special details

**Table:**

Geometry. All e.s.d.'s (except the e.s.d. in the dihedral angle between two l.s. planes) are estimated using the full covariance matrix. The cell e.s.d.'s are taken into account individually in the estimation of e.s.d.'s in distances, angles and torsion angles; correlations between e.s.d.'s in cell parameters are only used when they are defined by crystal symmetry. An approximate (isotropic) treatment of cell e.s.d.'s is used for estimating e.s.d.'s involving l.s. planes.
Refinement. Refinement of *F*^2^ against ALL reflections. The weighted *R*-factor *wR* and goodness of fit *S* are based on *F*^2^, conventional *R*-factors *R* are based on *F*, with *F* set to zero for negative *F*^2^. The threshold expression of *F*^2^ > σ(*F*^2^) is used only for calculating *R*-factors(gt) *etc*. and is not relevant to the choice of reflections for refinement. *R*-factors based on *F*^2^ are statistically about twice as large as those based on *F*, and *R*- factors based on ALL data will be even larger.

### Fractional atomic coordinates and isotropic or equivalent isotropic displacement parameters (Å^2^)

**Table:**

	*x*	*y*	*z*	*U*_iso_*/*U*_eq_	
Fe1	0.5000	0.5000	0.0000	0.0222 (2)	
Fe2	1.0000	0.5000	0.5000	0.0258 (2)	
C1	0.6446 (4)	0.3738 (3)	0.2523 (3)	0.0270 (6)	
C2	0.4924 (4)	0.2309 (3)	0.1667 (3)	0.0275 (6)	
C3	0.4514 (5)	0.0824 (3)	0.2147 (3)	0.0330 (7)	
H3	0.5126	0.0679	0.3050	0.040*	
C4	0.3189 (5)	−0.0438 (3)	0.1270 (3)	0.0331 (7)	
H4	0.2889	−0.1449	0.1579	0.040*	
C5	0.2302 (4)	−0.0216 (3)	−0.0065 (3)	0.0278 (6)	
C6	0.2767 (4)	0.1328 (3)	−0.0474 (3)	0.0279 (6)	
H6	0.2161	0.1496	−0.1372	0.033*	
C7	0.0905 (4)	−0.1587 (4)	−0.1093 (3)	0.0318 (7)	
N1	0.4039 (3)	0.2565 (3)	0.0369 (3)	0.0261 (5)	
O1	0.8315 (4)	0.6187 (3)	0.5803 (3)	0.0448 (6)	
H1A	0.7132	0.6145	0.5373	0.054*	
H1B	0.9150	0.7017	0.6279	0.054*	
O2	0.9411 (3)	0.3350 (3)	0.6494 (2)	0.0425 (6)	
H2B	0.8576	0.2419	0.6286	0.051*	
H2A	1.0460	0.3570	0.7156	0.051*	
O3	0.7348 (3)	0.3533 (2)	0.3701 (2)	0.0352 (5)	
O4	0.6748 (3)	0.5047 (2)	0.1970 (2)	0.0334 (5)	
O5	0.2787 (3)	0.5429 (3)	0.0954 (2)	0.0361 (5)	
H5A	0.1792	0.5354	0.0276	0.043*	
H5B	0.2288	0.4806	0.1537	0.043*	
O6	0.0097 (4)	−0.1287 (3)	−0.2262 (3)	0.0473 (6)	
O7	0.0653 (4)	−0.2939 (3)	−0.0688 (3)	0.0430 (6)	
O8	0.4663 (4)	0.2880 (4)	0.6019 (3)	0.0640 (8)	
H8B	0.4601	0.3105	0.6859	0.077*	
H8A	0.5871	0.3270	0.5904	0.077*	
O9	0.1591 (7)	−0.0327 (5)	0.4428 (6)	0.1112 (15)	
H9B	0.2489	0.0471	0.4912	0.133*	
H9A	0.1463	0.0205	0.3718	0.133*	

### Atomic displacement parameters (Å^2^)

**Table:**

	*U*^11^	*U*^22^	*U*^33^	*U*^12^	*U*^13^	*U*^23^
Fe1	0.0226 (3)	0.0153 (3)	0.0243 (3)	0.0050 (2)	−0.0034 (2)	−0.0004 (2)
Fe2	0.0269 (4)	0.0200 (3)	0.0247 (3)	0.0056 (2)	−0.0043 (2)	−0.0003 (2)
C1	0.0253 (14)	0.0248 (14)	0.0301 (15)	0.0088 (12)	0.0027 (11)	−0.0011 (11)
C2	0.0261 (14)	0.0260 (14)	0.0284 (15)	0.0082 (12)	0.0021 (11)	−0.0013 (11)
C3	0.0369 (17)	0.0282 (15)	0.0302 (16)	0.0083 (13)	0.0019 (13)	0.0059 (12)
C4	0.0357 (16)	0.0215 (14)	0.0403 (17)	0.0090 (13)	0.0041 (13)	0.0059 (12)
C5	0.0251 (14)	0.0225 (14)	0.0355 (16)	0.0079 (12)	0.0054 (12)	0.0003 (12)
C6	0.0277 (15)	0.0226 (13)	0.0314 (15)	0.0088 (12)	0.0006 (12)	−0.0004 (11)
C7	0.0242 (14)	0.0258 (15)	0.0432 (18)	0.0083 (12)	0.0023 (13)	−0.0049 (13)
N1	0.0259 (12)	0.0187 (11)	0.0308 (13)	0.0071 (10)	−0.0003 (10)	0.0014 (9)
O1	0.0403 (13)	0.0383 (13)	0.0492 (14)	0.0153 (11)	−0.0089 (11)	−0.0151 (11)
O2	0.0407 (13)	0.0349 (12)	0.0411 (13)	0.0033 (10)	−0.0026 (10)	0.0113 (10)
O3	0.0366 (12)	0.0278 (11)	0.0308 (11)	0.0047 (9)	−0.0072 (9)	0.0010 (8)
O4	0.0367 (12)	0.0228 (10)	0.0314 (11)	0.0071 (9)	−0.0096 (9)	−0.0011 (8)
O5	0.0320 (11)	0.0320 (12)	0.0439 (13)	0.0104 (10)	0.0072 (10)	0.0046 (10)
O6	0.0572 (15)	0.0302 (12)	0.0434 (14)	0.0136 (11)	−0.0134 (11)	−0.0088 (10)
O7	0.0367 (13)	0.0198 (11)	0.0632 (16)	0.0040 (10)	−0.0020 (11)	−0.0008 (10)
O8	0.0469 (16)	0.075 (2)	0.066 (2)	0.0196 (16)	0.0038 (14)	0.0019 (16)
O9	0.116 (3)	0.054 (2)	0.163 (5)	0.019 (2)	0.041 (3)	0.000 (2)

### Geometric parameters (Å, °)

**Table:**

Fe1—O4	2.058 (2)	C4—C5	1.372 (4)
Fe1—O4^i^	2.058 (2)	C4—H4	0.9300
Fe1—O5^i^	2.100 (2)	C5—C6	1.397 (4)
Fe1—O5	2.100 (2)	C5—C7	1.515 (4)
Fe1—N1	2.125 (2)	C6—N1	1.328 (4)
Fe1—N1^i^	2.125 (2)	C6—H6	0.9300
Fe2—O1	2.071 (2)	C7—O6	1.241 (4)
Fe2—O1^ii^	2.071 (2)	C7—O7	1.245 (4)
Fe2—O3^ii^	2.080 (2)	O1—H1A	0.8499
Fe2—O3	2.080 (2)	O1—H1B	0.8499
Fe2—O2	2.092 (2)	O2—H2B	0.8500
Fe2—O2^ii^	2.092 (2)	O2—H2A	0.8501
C1—O3	1.242 (3)	O5—H5A	0.8499
C1—O4	1.268 (4)	O5—H5B	0.8500
C1—C2	1.496 (4)	O8—H8B	0.8500
C2—N1	1.351 (4)	O8—H8A	0.8499
C2—C3	1.376 (4)	O9—H9B	0.8499
C3—C4	1.368 (4)	O9—H9A	0.8500
C3—H3	0.9300		
			
O4—Fe1—O4^i^	180.00 (11)	N1—C2—C1	115.5 (2)
O4—Fe1—O5^i^	90.89 (9)	C3—C2—C1	122.3 (3)
O4^i^—Fe1—O5^i^	89.11 (9)	C4—C3—C2	118.8 (3)
O4—Fe1—O5	89.11 (9)	C4—C3—H3	120.6
O4^i^—Fe1—O5	90.89 (9)	C2—C3—H3	120.6
O5^i^—Fe1—O5	180.00 (6)	C3—C4—C5	120.2 (3)
O4—Fe1—N1	79.00 (8)	C3—C4—H4	119.9
O4^i^—Fe1—N1	101.00 (8)	C5—C4—H4	119.9
O5^i^—Fe1—N1	88.20 (9)	C4—C5—C6	117.9 (3)
O5—Fe1—N1	91.80 (9)	C4—C5—C7	121.9 (3)
O4—Fe1—N1^i^	101.00 (8)	C6—C5—C7	120.2 (3)
O4^i^—Fe1—N1^i^	79.00 (8)	N1—C6—C5	122.5 (3)
O5^i^—Fe1—N1^i^	91.80 (9)	N1—C6—H6	118.7
O5—Fe1—N1^i^	88.20 (9)	C5—C6—H6	118.7
N1—Fe1—N1^i^	180.0	O6—C7—O7	125.1 (3)
O1—Fe2—O1^ii^	180.00 (12)	O6—C7—C5	118.1 (3)
O1—Fe2—O3^ii^	90.59 (9)	O7—C7—C5	116.8 (3)
O1^ii^—Fe2—O3^ii^	89.41 (9)	C6—N1—C2	118.4 (2)
O1—Fe2—O3	89.41 (9)	C6—N1—Fe1	129.8 (2)
O1^ii^—Fe2—O3	90.59 (9)	C2—N1—Fe1	111.81 (18)
O3^ii^—Fe2—O3	180.0	Fe2—O1—H1A	122.3
O1—Fe2—O2	89.15 (10)	Fe2—O1—H1B	107.1
O1^ii^—Fe2—O2	90.85 (10)	H1A—O1—H1B	122.4
O3^ii^—Fe2—O2	93.74 (9)	Fe2—O2—H2B	122.9
O3—Fe2—O2	86.26 (9)	Fe2—O2—H2A	108.0
O1—Fe2—O2^ii^	90.85 (10)	H2B—O2—H2A	122.9
O1^ii^—Fe2—O2^ii^	89.15 (10)	C1—O3—Fe2	129.74 (18)
O3^ii^—Fe2—O2^ii^	86.26 (9)	C1—O4—Fe1	116.24 (18)
O3—Fe2—O2^ii^	93.74 (9)	Fe1—O5—H5A	104.8
O2—Fe2—O2^ii^	180.0	Fe1—O5—H5B	119.7
O3—C1—O4	125.5 (3)	H5A—O5—H5B	105.0
O3—C1—C2	117.1 (2)	H8B—O8—H8A	110.3
O4—C1—C2	117.3 (2)	H9B—O9—H9A	91.5
N1—C2—C3	122.2 (3)		

Symmetry codes: (i) −*x*+1, −*y*+1, −*z*; (ii) −*x*+2, −*y*+1, −*z*+1.

### Hydrogen-bond geometry (Å, °)

**Table:**

*D*—H···*A*	*D*—H	H···*A*	*D*···*A*	*D*—H···*A*
O9—H9B···O8	0.85	2.32	3.159 (6)	171
O9—H9A···O6^iii^	0.85	2.06	2.849 (5)	154
O8—H8B···O5^iv^	0.85	2.55	3.204 (4)	134
O8—H8B···O4^iv^	0.85	2.51	3.171 (4)	136
O8—H8A···O3	0.85	2.55	3.177 (4)	132
O8—H8A···O2	0.85	2.44	3.201 (4)	149
O5—H5B···O7^iii^	0.85	2.22	2.706 (3)	116
O2—H2B···O9^v^	0.85	1.94	2.657 (5)	141
O2—H2A···O4^ii^	0.85	1.99	2.758 (3)	150
O1—H1B···O6^vi^	0.85	1.92	2.715 (3)	156
O1—H1A···O8^iv^	0.85	2.06	2.822 (4)	148

Symmetry codes: (iii) −*x*, −*y*, −*z*; (iv) −*x*+1, −*y*+1, −*z*+1; (v) −*x*+1, −*y*, −*z*+1; (ii) −*x*+2, −*y*+1, −*z*+1; (vi) *x*+1, *y*+1, *z*+1.
